# Three-Dimensional Amide Proton Transfer-Weighted Imaging for Differentiating between Glioblastoma, IDH-Wildtype and Primary Central Nervous System Lymphoma

**DOI:** 10.3390/cancers15030952

**Published:** 2023-02-02

**Authors:** Shigeo Ohba, Kazuhiro Murayama, Takao Teranishi, Masanobu Kumon, Shunsuke Nakae, Masao Yui, Kaori Yamamoto, Seiji Yamada, Masato Abe, Mitsuhiro Hasegawa, Yuichi Hirose

**Affiliations:** 1 Department of Neurosurgery, Fujita Health University School of Medicine, Toyoake 470-1192, Aichi, Japan; 2 Department of Radiology, Fujita Health University School of Medicine, Toyoake 470-1192, Aichi, Japan; 3 Canon Medical Systems Corporation, Otawara 324-8550, Tochigi, Japan; 4 Department of Diagnostic Pathology, Fujita Health University School of Medicine, Toyoake 470-1192, Aichi, Japan; 5 Department of Pathology, Fujita Health University School of Health Sciences, Toyoake 470-1192, Aichi, Japan

**Keywords:** APTw, CEST, glioblastoma, MIB-1 index, PCNSL

## Abstract

**Simple Summary:**

Although primary central nervous system lymphoma (PCNSL) is sometimes indistinguishable from glioblastoma, isocitrate dehydrogenase (IDH)-wildtype, the role of operation on them is very different; therefore, accurate preoperative diagnosis is crucial. In this study, we evaluated whether amide proton transfer-weighted (APTw) imaging was useful to distinguish PCNSL from glioblastoma, IDH-wildtype. The mean APTw signal value did not significantly differ between them; however, the percentile values from the 1st percentile to the 20th percentile APTw signals and the width_1–100_ APTw signals between the two diseases were significantly different. The area under the curve, sensitivity, and specificity were 0.796, 64.3%, and 88.9%, respectively, using the width_1–100_ APTw signal values. APTw imaging was useful to differentiate between PCNSL and glioblastoma, IDH-wildtype. Performing APTw imaging is recommended in cases in which PCNSL is one of the differential diagnoses.

**Abstract:**

Distinguishing primary central nervous system lymphoma (PCNSL) from glioblastoma, isocitrate dehydrogenase (IDH)-wildtype is sometimes hard. Because the role of operation on them varies, accurate preoperative diagnosis is crucial. In this study, we evaluated whether a specific kind of chemical exchange saturation transfer imaging, i.e., amide proton transfer-weighted (APTw) imaging, was useful to distinguish PCNSL from glioblastoma, IDH-wildtype. A total of 14 PCNSL and 27 glioblastoma, IDH-wildtype cases were evaluated. There was no significant difference in the mean APTw signal values between the two groups. However, the percentile values from the 1st percentile to the 20th percentile APTw signals and the width_1–100_ APTw signals significantly differed. The highest area under the curve was 0.796, which was obtained from the width_1–100_ APTw signal values. The sensitivity and specificity values were 64.3% and 88.9%, respectively. APTw imaging was useful to distinguish PCNSL from glioblastoma, IDH-wildtype. To avoid unnecessary aggressive surgical resection, APTw imaging is recommended for cases in which PCNSL is one of the differential diagnoses.

## 1. Introduction

Gliomas are the most common intra-axial primary brain tumors. Recently, isocitrate dehydrogenase (IDH) mutation was found to be associated with tumorigenesis, and gliomas have been classified into IDH-mutant and IDH-wildtype gliomas [1,2,3]. Based on the 2021 WHO Classification of Tumors of the Central Nervous System [4], the term “glioblastoma” was used for IDH-wildtype glioma. Glioblastoma, IDH-wildtype is among the most aggressive brain tumors, and its standard treatment is maximum safe resection followed by radiotherapy and chemotherapy [5]. The extent of resection or residual tumor volume has been reported to be associated with the prognosis in glioblastoma patients [6,7]. Recently, the effect of resection beyond the contrast-enhanced area on overall survival has been reported [8,9].

Conversely, primary central nervous system lymphoma (PCNLS)—a representative malignant intra-axial brain tumor—has also been reported, although it is less common. The effect of surgical resection on PCNSL is still controversial, and a biopsy is usually performed to diagnose PCNSL because it is sensitive to chemotherapy and/or radiotherapy [10,11]. The findings of conventional magnetic resonance imaging (MRI) of glioblastomas and malignant lymphomas are often similar, and distinguishing them from one another is sometimes very difficult [12,13,14]. Given that the role of operation on them is very different, accurate preoperative diagnosis is crucial.

Amide proton transfer-weighted (APTw) imaging is a specific type of chemical exchange saturation transfer (CEST) imaging modality that can indirectly measure the endogenous mobile proteins and peptides in tissues [15]. APTw imaging has been used for various diseases, such as brain tumors, several types of cancer, and stroke [16,17,18,19]. Regarding brain tumors, several types—such as glioma, meningioma, lymphoma, metastatic tumors, and schwannoma—have been investigated [20,21,22]. Moreover, molecular biomarkers of gliomas [23,24] and their treatment response [25,26] have been studied.

No paper has been published to show the usefulness of APTw to distinguish PCNSL from glioblastoma, IDH-wildtype, although one paper was published where PCNSL and high-grade glioma were differentiated using APTw [20]. In this report, we aimed to evaluate whether APTw imaging is useful to distinguish PCNSL from glioblastoma, IDH-wildtype.

## 2. Materials and Methods

### 2.1. Patients

This retrospective study was approved by the ethics review committee of Fujita Health University. The study enrolled 41 consecutive patients with histopathologically defined PCNSL or glioblastoma, IDH-wildtype who underwent APTw imaging before their initial surgery.

### 2.2. MRI Protocol and Image Analysis

All MR examinations were performed with a 3-T MR system (Vantage Centurian, Canon Medical Systems Corporation, Otawara, Japan) using a 32-channel head coil. MR acquisition, reconstruction, and fully automated post-processing of APTw images were performed with non-commercial CEST analysis software (v.6-8) on a 3-T MR system. APTw images were acquired on the axial plane by three-dimensional (3D) fast field echo (FFE) sequences with one sinc-shaped magnetization transfer (MT) pulse per segment. For 3D APTw imaging, the nominal B1rm of each MT pulse was set to 2 (uT). Thirty-one frequency offsets of the MT pulses were used for 3D APTw imaging, with an offset unevenly spaced between −5.8 and +5.8 ppm. Signal reference images (S0) were also acquired using each sequence without MT pulses to estimate the MT ratio (MTR), expressed as shown in the following formula: MTR = 1 − S(ω)/S0, where ω is the frequency offset in ppm. The other imaging parameters for the 3D APTw image were set as follows: field of view, 250 (mm); 128 × 128 acquisition matrix; 256 × 256 reconstruction matrix; 5 mm slice thickness; parallel imaging factor of 2.0 in the phase-encoding direction; TR/TE, 4.0 (ms)/1.6 (ms); number of slices, 30; and number of segments, 4.

**Figure 1:**
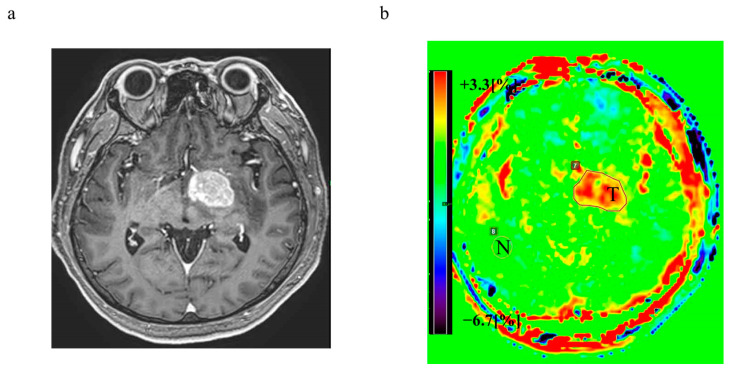
Representative (**a**) T1-weighted image with gadolinium and (**b**) ATPw image. T: tumor, N: normal tissue; ATPw image: amide proton transfer-weighted image.

After each of the CEST data for all patients had been obtained, a pixel-by-pixel MTR asymmetry (MTR_asym_) calculation at 3.5 ppm was automatically performed. The region of interest (ROI) was placed on the defined margins with contrast enhancement on the contrast-enhanced T1-weighted image (Figure 1). The magnetization transfer asymmetry value at 3.5 ppm (MTR_asym_ at 3.5 ppm) based on the histogram analysis within the ROI was computationally determined on 3D APTw images, and the APTw signal (%) was calculated from the z-spectra for each pixel using the following formula:

APTw signal contrast between the lesion and normal-appearing white matter (NAWM) (%) = {MTR_asym_ (lesion at 3.5 ppm) − MTR_asym_ (NAWM at 3.5 ppm)} × 100

Contrast-enhanced T1-weighted 3D FFE was acquired with the following parameters: TR 7.9 ms/TE 3.7 ms; flip angle, 20 degree; pixel size, 0.5 × 0.5 mm^2^; reconstruction matrix, 512 × 512; field of view, 256 × 256 mm; slice thickness, 1 mm; number of slices, 180; parallel imaging factor, 2.0 in the phase-encoding direction; and number of excitations, 1. The contrast-enhanced T1-weighted 3D FFE was obtained after administration of a standard dose of a contrast medium (0.1 mmol/kg body weight).

### 2.3. Histopathology

Histopathological diagnosis was reevaluated based on the 2021 WHO classification. The MIB-1 index was calculated as the percentage of immunolabeled tumor cell nuclei for anti-Ki67 antibody per total tumor cell nuclei. Immunohistochemistry was performed using p53, CD10, CD20, bcl-6, and MUM1 antibodies. PCNSL was subclassified as germinal center B-cell (GCB) and non-GCB subtypes based on the immunohistochemical assay results [27].

### 2.4. DNA Isolation and Comparative Genomic Hybridization and PCR-Based Sequencing of the IDH1 or IDH2 Genes

Deoxyribonucleic acid (DNA) preparation from microdissected tissue and polymerase chain reaction (PCR)-based sequencing of the IDH1 or IDH2 genes was performed as described previously [28].

### 2.5. Methylation-Specific PCR of the MGMT Gene

Tumor DNA was extracted from microdissected tissue and analyzed after bisulfite conversion using primers for methylated and unmethylated sequences, using the EZ DNA Methylation-Direct Kit (Zymo Research Corp., Orange, CA, USA) as described previously [29].

### 2.6. Statistical Analyses 

The mean and percentile APTw signals were compared using *t*-tests to analyze the statistical differences between PCNSL and glioblastoma, IDH-wildtype. Receiver operating characteristic (ROC) curve analysis was performed to determine the optimal cutoff value for the best discriminating APTw signal to differentiate between PCNSL and glioblastoma, IDH-wildtype. The optimal threshold value for the APTw signal was estimated by maximizing the Youden index (sensitivity + specificity − 1). All statistical analyses were performed with JMP 13 (SAS Institute Inc. Japan, Tokyo, Japan). A *p*-value < 0.05 was considered significant in this study.

## 3. Results

### 3.1. The Clinical Characteristics of PCNSL and Glioblastoma, IDH-Wildtype

Altogether, 41 cases were evaluated; 29 cases were male, and 14 cases were female. The age varied from 19 to 87 years. A total of 27 cases were glioblastoma, IDH-wildtype, whereas 14 cases were PCNSL The mean APTw signal of PCNSL and glioblastoma, IDH-wildtype was 2.3 8 ± 0.79 and 2.25 ± 0.58, respectively (Table 1).

### 3.2. APTw Imaging Was Useful to Distinguish PCNSL from Glioblastoma, IDH-Wildtype

**Figure 2:**
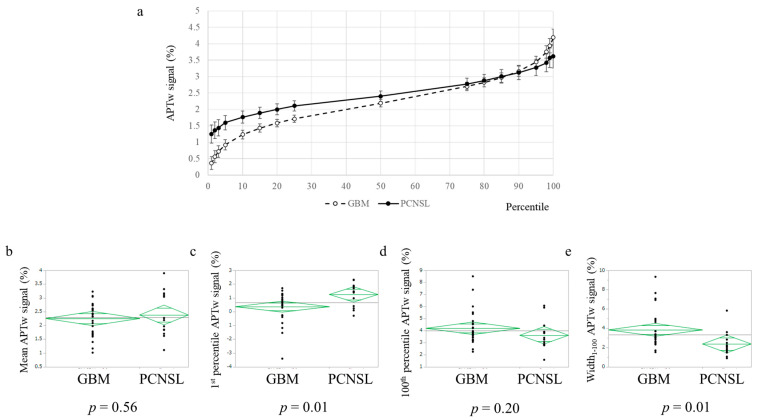
Each percentile APTw signal of PCNSL and glioblastoma, IDH-wildtype. (**a**) Graph of each percentile APTw signal of PCNSL and glioblastoma, IDH-wildtype; error bars: standard error. (**b**) Mean, (**c**) 1st percentile, (**d**) 100th percentile, and (**e**) width_1–100_ APTw signals of glioblastoma, IDH-wildtype and PCNSL.

**Figure 3:**
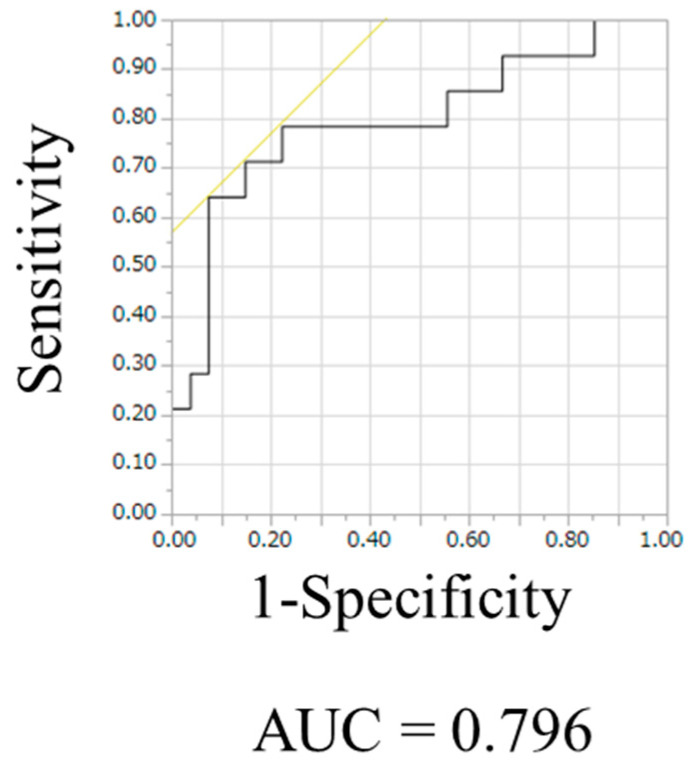
ROC of the width_1–100_ APTw signal, showing an AUC of 0.796.

The mean values and percentiles of the APTw signals are summarized in Table 2. The graph of the percentiles of the APTw signal is shown in Figure 2. There were no significant differences in the mean values of the APTw signals between the PCNSL and glioblastoma, IDH-wildtype cases (*p* = 0.56). Conversely, the percentile from the 1st percentile to the 20th percentile of the APTw signals was significantly different between the PCNSL and glioblastoma, IDH-wildtype cases (*p* < 0.05). The width_1–100_ (100th percentile–1st percentile) APTw signal values in the PCNSL group were significantly lower than those of the glioblastoma, IDH-wildtype group (*p* < 0.05). If a statistical difference in the APTw signal values was observed between the PCNSL and glioblastoma, IDH-wildtype cases, ROC analysis was performed, and the area under the curve (AUC) was calculated. The highest AUC value was 0.796, which was obtained when the width_1–100_ APTw signal value was used for the ROC curve analysis (Figure 3). The cutoff value was 2.23. The sensitivity and specificity values were 64.3% and 88.9%, respectively.

### 3.3. The Relationship between MIB-1 Index and APTw in Glioblastoma, IDH-Wildtype and PCNSL Cases

**Figure 4:**
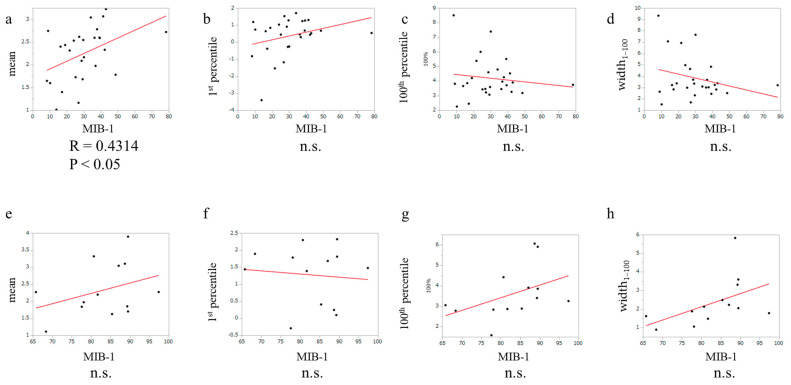
The correlation between the MIB1 index and the mean, 1st percentile, 100th percentile, and width_1–100_ APTw signals of (**a**–**d**) glioblastoma, IDH-wildtype and (**e**–**h**) PCNSL.

Correlation has been reported between the MIB-1 index and APTw signal in glioma [16]. To evaluate whether the MIB-1 index was correlated with the APTw signal in only glioblastoma, IDH-wildtype, a simple linear regression test was performed. There was no correlation between the MIB-1 index and the 1st and 100th percentile APTw signals or the width_1–100_ APTw signal, whereas the mean APTw signal was moderately correlated with the MIB-1 index (Figure 4a–d). For PCNSL, there was no correlation between the MIB-1 index and the mean, 1st and 100th percentile, or width_1–100_ APTw signals (Figure 4e–h).

### 3.4. The Correlation between Molecular Markers and APTw Signals in PCNLS

Based on the APTw signal, the cases were divided into predictable and unpredictable diseases. When a histopathological diagnosis was consistent with a prediction based on the width_1–100_ APTw signal, we characterized the case as predictable in this study. To investigate whether a subtype of PCNSL (i.e., GCB or non-GCB) was associated with a predictable diagnosis, or whether the MIB-1 index was different between the predictable and unpredictable PCNSL cases, an immunohistological analysis was performed. The PCNSL subtype was not correlated with predictable or unpredictable disease (Table 3). There were no statistical differences in the mean, 1st and 100th percentile, and width_1–100_ APTw signals between the GCB and non-GCB types (Figure S1).

### 3.5. The Correlation between Molecular Markers and APTw in Glioblastoma, IDH-Wildtype Cases 

To detect any molecular marker as being associated with predictable disease diagnosis in glioblastoma, IDH-wildtype cases, an immunohistological analysis of p53 and Ki-67 was performed, along with an investigation of the status of the MGMT promoter. There was no correlation between predictable diagnosis and p53 immunopositivity, MIB-1 index, or MGMT promoter methylation status (Table 4). There were no statistical differences in the mean, 1st and 100th percentile, or width_1–100_ APTw signals between the p53-positive and p53-negative tumors, nor between methylated and unmethylated MGMT promoter tumors (Figures S2 and S3).

### 3.6. Representative Cases

**Figure 5:**
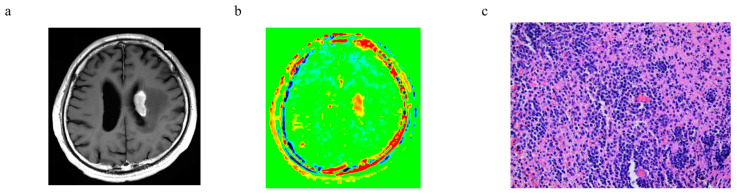
A representative case of PCNSL: (**a**) T1-weighted image with gadolinium, (**b**) APTw image, and (**c**) histology (H–E staining).

**Figure 6:**
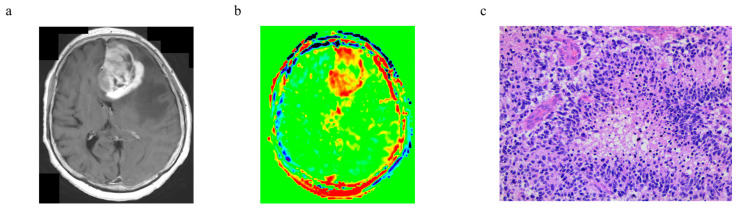
A representative case of glioblastoma, IDH-wildtype: (**a**) T1-weighted image with gadolinium, (**b**) APTw image, and (**c**) histology (H–E staining).

The representative cases of PCNSL and glioblastoma, IDH-wildtype are shown in Figure 5 and Figure 6, respectively.

## 4. Discussion

Although PCNSL and glioblastoma, IDH-wildtype sometimes show a similar appearance on conventional MRI, the development of new methods that can differentiate them is expected.

Glioma is the most common intra-axial brain tumor. After the discovery of the IDH mutation, which is considered to be the driving mutation for glioma-harboring IDH mutants, gliomas were divided into IDH-mutant and IDH-wildtype subtypes. IDH-mutant gliomas are further subclassified into astrocytoma, IDH-mutant and oligodendroglioma, IDH-mutant and 1p/19q-codeleted [1,2,3]. Some studies have reported on the differences in amino-weighted CEST contrast values between IDH-mutant and IDH-wildtype gliomas [23,30,31,32], between astrocytomas and oligodendrogliomas [33], and among the tumors of different WHO classification grades [34,35]. Based on the Classification of Tumors of the Central Nervous System published in 2021, glioblastoma is not the name given to gliomas harboring mutant IDH [4]. Therefore, we reevaluated glioblastoma based on the 2021 WHO Classification of Tumors of the Central Nervous System, and only glioblastoma, IDH-wildtype cases were included in the study.

The usefulness of several methods—such as magnetic resonance spectroscopy (MRS), perfusion MRI, and positron emission tomography CT—to distinguish PCNSL from glioblastoma has been reported [36,37,38]. In the present study, we evaluated the usefulness of APTw imaging to differentiate PCNSL and glioblastoma, IDH-wildtype. APTw imaging is a new method with several advantages, as follows: (1) It does not need contrast medium administration. For some special methods that have been reported to be useful for distinguishing PCNSL from glioblastoma, such as perfusion MRI, a contrast medium is needed for the imaging [36]. (2) It has a short examination time. We previously reported the usefulness of MRS to differentiate PCNSL and glioblastoma; however, performing MRS takes a long time, which is sometimes difficult for patients with poor conditions [37]. (3) APTw imaging can be performed in 3D, and the data can be analyzed at any time after the examination [39,40]. The ROI can be defined after the examination, as opposed to other imaging modalities. Therefore, if the usefulness of APTw imaging to differentiate PCNSL and glioblastoma is demonstrated, it can be considered the optimal method for differentiating brain tumors.

One study showed that APTw imaging is valuable, as it could identify PCNSs and high-grade gliomas [20]. This previous study calculated the maximum APTw intensity (APTw_max_), minimum APTw intensity (APTw_min_), APTw signal heterogeneity within tumor cores (APTw_max−min_), and mean APTw signals. The AUC value of APTw_max−min_ was the highest among the abovementioned parameters [20]. In our study, after placing the ROI on the defined margins with contrast enhancement, percentile APTw signals were measured automatically. The lower percentile APTw signal of the glioblastoma, IDH-wildtype group was significantly lower than that of the PCNSL group, while the higher percentile APTw signal of the glioblastoma, IDH-wildtype group tended to be higher than that of the PCNSL group. These results are considered to be due to the intratumoral heterogeneity of glioblastoma, IDH-wildtype and the homogeneity of PCNSL. Consistent with the previous study, our study demonstrated width_1–100_ APTw signals that were significantly different between the PCNSL and glioblastoma, IDH-wildtype groups, showing the highest AUC values. Although several previous studies used 2D APTw imaging, 3D APTw imaging sequences were used in our study. Compared to 2D APTw imaging, 3D APTw imaging takes less time; hence, more slices were taken, and the appropriate slices were able to be selected from them. In many studies, the ROI was placed manually [19,20,21,23]. To exclude the intentional placement of the ROI, we circled the enhanced area of the tumor and automatically calculated the percentile APTw signals. Because of intratumoral heterogeneity of glioblastoma, IDH-wildtype, we used histogram analysis after setting the ROI in this study. These evaluations can lead to a more objective comparison between PCNSL and glioblastoma, IDH-wildtype.

Regarding the MIB-1 index, Togao et al. found that there was a moderate positive correlation between the APTw signal intensity and the Ki-67 labeling index [19]. In their study, grade 2–4 gliomas were included. They also showed the differences in APTw intensity signals among tumors of different grades. In our study, where only glioblastoma, IDH-wildtype cases were included, the mean APTw signal was also moderately correlated with the MIB-1 index. Regarding the correlation between APTw signal and MGMT promoter status, the findings of previous reports were controversial. Jiang et al. reported that the mean, variance, 50th percentile, 90th percentile, and width_10–90_ APTw values were statistically different between the MGMT unmethylated and methylated glioblastomas [41]. Conversely, other studies reported no differences in the APTw signals between the MGMT unmethylated and methylated glioblastomas, which was consistent with our study’s findings [23,32]. Based on the values of the APTw signals and diagnosis, each case was classified as predictable or unpredictable. Comparing the predictable and unpredictable cases of PCNSL, we did not find any differences in the subtypes or MIB-1 index. The p53 expression, MIB-1 index, and MGMT promoter methylation status showed no significant differences between the predictable and unpredictable glioblastoma, IDH-wildtype cases. Further investigation with more cases might reveal differences between the predictable and unpredictable cases as evaluated by using APTw signals.

We highlighted three points in our study: First, we compared the data of PCNSL with only glioblastoma, IDH-wildtype, excluding other types of glioma. Second, instead of 2D, 3D APTw imaging was used for analysis. Finally, the ROI was placed conforming to enhanced lesions, and each percentile APTw signal was calculated automatically. Then, each percentile of the APTw signals, including the mean and width_1–100_ APTw signals, was compared between the PCNSL and glioblastoma, IDH-wildtype cases. The results analyzed based on these methods were considered to be reliable and significant.

### Limitations

This study has several limitations. This study was retrospective, and the number of included cases was low. However, considering the rarity of brain tumors, as well as the more objective evaluation of APTw signals, our study’s results are believed to be informative for the readers.

## 5. Conclusions

APTw imaging was useful to distinguish PCNSL from glioblastoma, IDH-wildtype. Given that a biopsy procedure is sufficient for PCNSLs as a surgical treatment method, to avoid unnecessary aggressive surgical resection, it is worth performing APTw imaging in cases where PCNSL is considered as one of the differential diagnoses.

## Figures and Tables

**Table 1:** Clinical characteristics of PCNSL and glioblastoma, IDH-wildtype.

		PCNCL	Glioblastoma, IDH-Wildtype	*p*-Value
Number		14	27	
Age (mean ± standard deviation)		66.3 ± 11.1	65.0 ± 17.9	n.s.
Gender	M	8	21	n.s.
	F	6	6	
Mean APTw signal (mean ± standard deviation)		2.38 ± 0.79	2.25 ± 0.58	n.s.

**Table 2:** The mean, percentile, and width_1–100_ APTw signal values of PCNSL and glioblastoma, IDH-wildtype.

		Percentile																		
		1	2	3	5	10	15	20	25	50	75	80	85	90	95	98	99	100	Width_1–100_	Mean
APTw signal (%)	PCNSL	1.25	1.37	1.44	1.60	1.77	1.89	2.00	2.11	2.40	2.78	2.88	3.01	3.12	3.28	3.42	3.57	3.62	2.37	2.38
	GBM	0.36	0.56	0.72	0.92	1.24	1.43	1.59	1.72	2.19	2.70	2.83	2.97	3.16	3.45	3.75	3.94	4.19	3.83	2.25
*p*-value		0.01	0.02	0.02	0.02	0.02	0.04	0.05	0.05	0.29	0.73	0.81	0.88	0.88	0.58	0.33	0.32	0.20	0.01	0.56
Cutoff value		1.39	1.47	1.51	1.57	1.74	1.79	1.86											2.23	
AUC		0.75	0.76	0.76	0.78	0.70	0.70	0.67											0.80	

**Table 3:** The subtype and MIB-1 index of predictable and unpredictable PCNSLs.

	Predictable	Unpredictable	*p*-Value
Number	9	5	
GCB	3	2	1.00
Non-GCB	5	3	
MIB-1 index (mean ± standard deviation)	80.7 ± 9.9	88.2 ± 1.9	0.17

**Table 4:** The status of p53, MGMT promoter, and MIB-1 index in predictable and unpredictable glioblastoma, IDH-wildtype cases.

		Predictable	Unpredictable	*p*-Value
Number		24	3	
p53	Positive	16	2	1.00
	Negative	8	1	
MGMT promoter	Methylated	5	2	0.18
	Unmethylated	9	0	
MIB-1 index (mean ± standard deviation)		29.1 ± 11.2	38.6 ± 35.5	0.30

## Data Availability

The data used in this study are available from the corresponding authors upon reasonable request.

## Supplementary Materials

The following supporting information can be downloaded at: https://www.mdpi.com/article/10.3390/cancers15030952/s1, Figure S1: The mean, 1st percentile, 100th percentile, and width_1–100_ APTw signals of the GCB and non-GCB types of PCNSL; Figure S2: The mean, 1st percentile, 100th percentile, and width_1–100_ APTw signals of immunohistochemically p53-positive and -negative glioblastoma, IDH-wildtype; Figure S3: The mean, 1st percentile, 100th percentile, and width_1–100_ APTw signals of MGMT promoter methylated and unmethylated glioblastoma, IDH-wildtype.
