# Supplementary figures and images for: Three-Dimensional Amide Proton Transfer-Weighted Imaging for Differentiating between Glioblastoma, IDH-Wildtype and Primary Central Nervous System Lymphoma
Source: Cancers (Basel). 2023 Feb 2;15(3):952. doi: 10.3390/cancers15030952 (PMC9913574; doi:10.3390/cancers15030952)

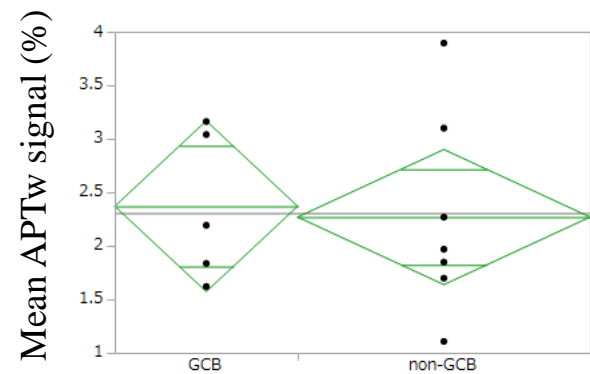

P=0.83

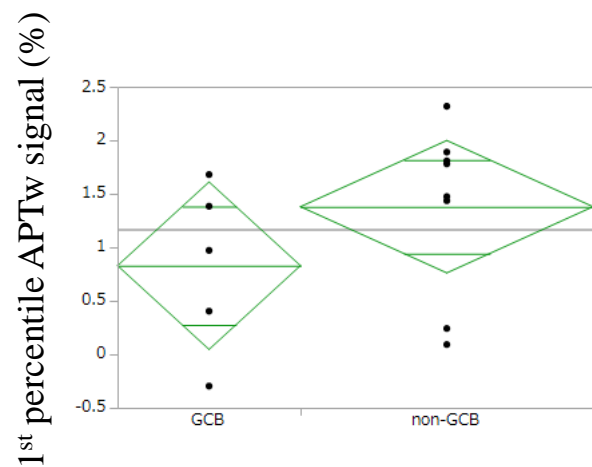

P=0.25

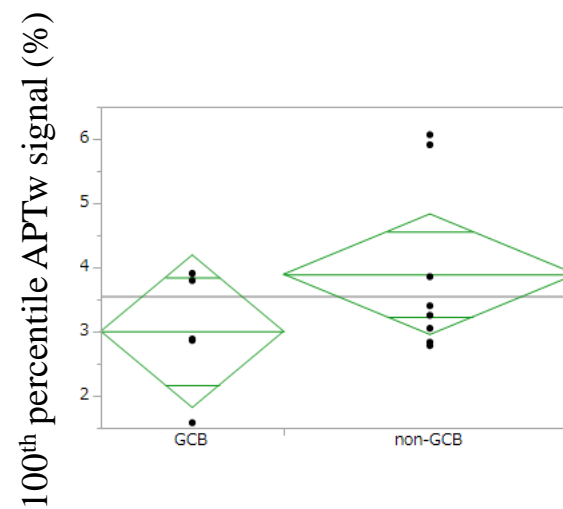

P=0.22

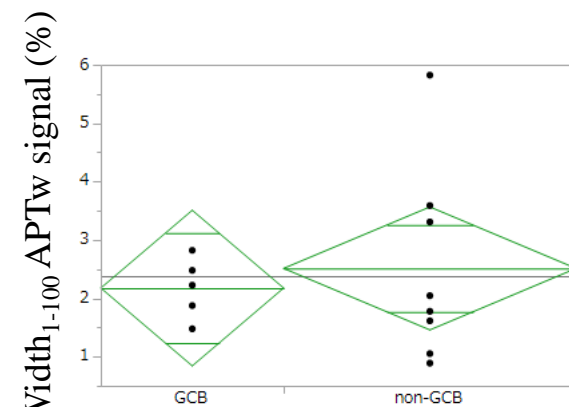

P=0.67

Supplementary Figure 1

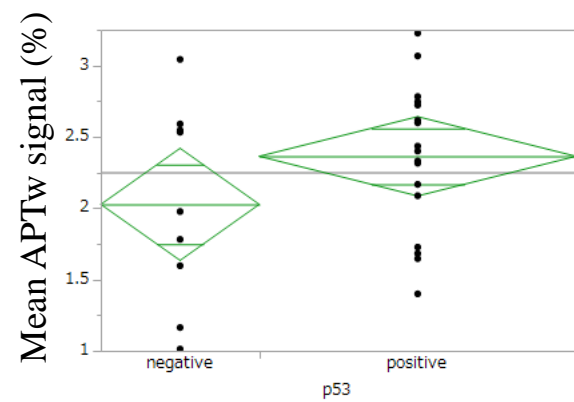

P=0.16

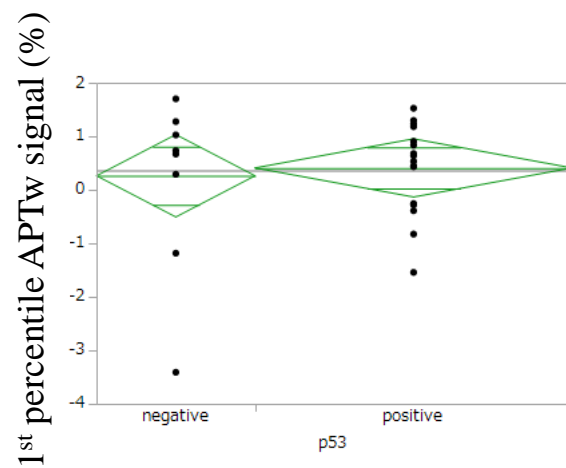

P=0.75

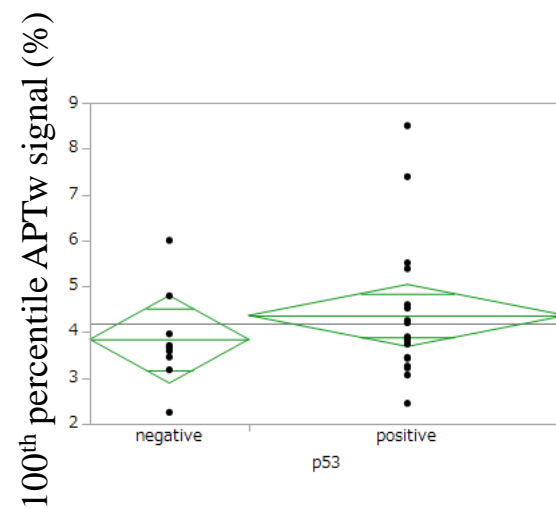

P=0.37

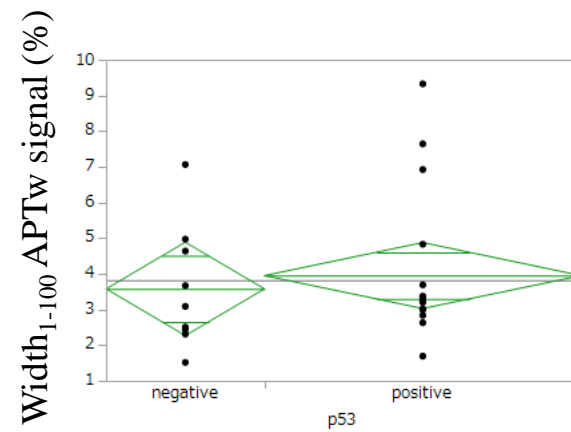

P=0.64

Supplementary Figure 2

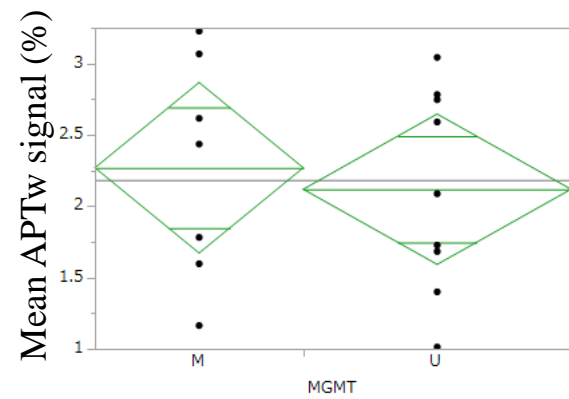

P=0.69

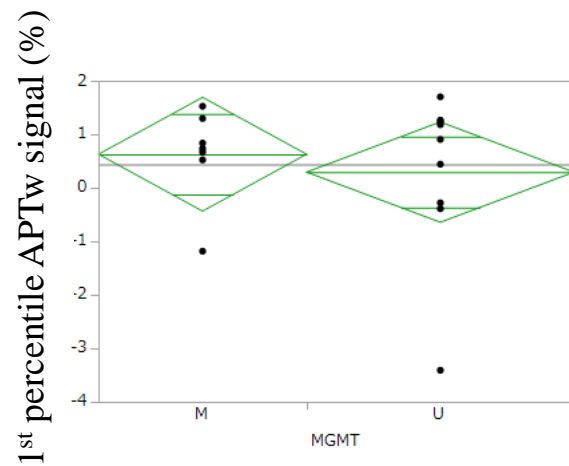

P=0.62

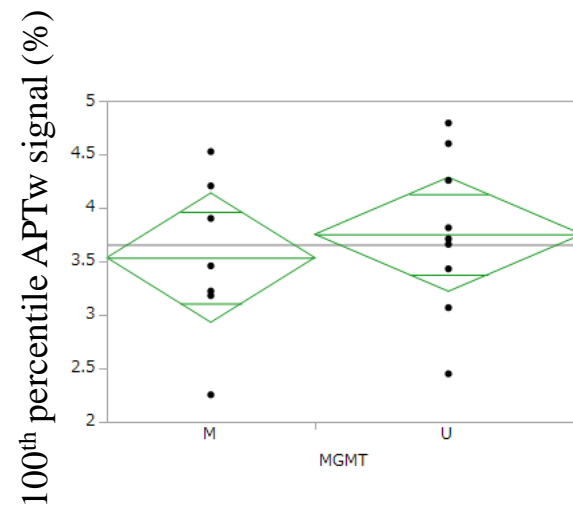

P=0.57

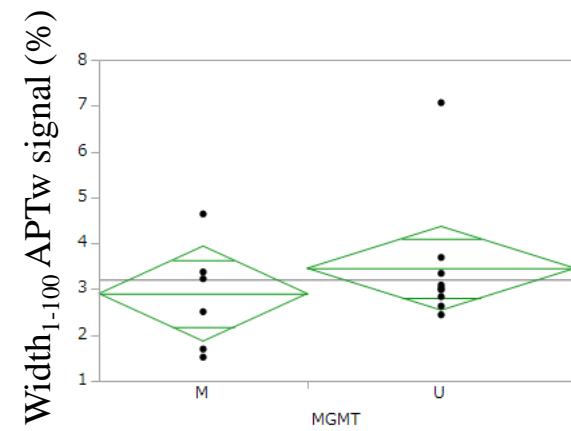

P=0.41

Supplementary Figure 3

Supplement: Supplementary file 1 [file cancers-15-00952-s001.zip › cancers-2116282-supplementary.pdf]
